# Digitomotography in Parkinson’s Disease: A Cross-Sectional and Longitudinal Study

**DOI:** 10.1371/journal.pone.0123914

**Published:** 2015-04-22

**Authors:** Walter Maetzler, Maren Ellerbrock, Tanja Heger, Christian Sass, Daniela Berg, Ralf Reilmann

**Affiliations:** 1 Hertie Institute for Clinical Brain Research, Department of Neurodegeneration, Center of Neurology, University of Tuebingen, Tuebingen, Germany; 2 DZNE, German Center for Neurodegenerative Diseases, Tuebingen, Germany; 3 Clinical Center Lunenburg, Clinic of Neurology, Lunenburg, Germany; 4 George-Huntington-Institute, Technology-Park Muenster, Muenster, Germany; 5 Department of Radiology, University of Muenster, Muenster, Germany; University of Medicine & Dentistry of NJ - New Jersey Medical School, UNITED STATES

## Abstract

Motor symptoms in Parkinson’s disease (PD) are usually assessed with semi-quantitative tests such as the Unified PD Rating Scale (UPDRS) which are limited by subjectivity, categorical design, and low sensitivity. Particularly bradykinesia as assessed e.g. with speeded index finger tapping exhibits low validity measures. This exploratory study set out to (i) assess whether force transducer-based objective and quantitative analysis of motor coordination in index finger tapping is able to distinguish between PD patients and controls, and (ii) assess longitudinal changes. Sixteen early-stage and 17 mid-stage PD patients as well as 18 controls were included in the cross-sectional part of the study; thirteen, 16 and 16 individuals of the respective groups agreed in a reassessment 12 months later. Frequency, force, rhythmicity, regularity and laterality of speeded and metronome paced tapping were recorded by digitomotography using a quantitative motor system ("Q-Motor"). Analysis of cross-sectional data revealed most consistent differences between PD patients and controls in variability of tap performance across modalities assessed. Among PD patients, variability of taps and the ability to keep a given rhythm were associated with UPDRS motor and finger tapping scores. After 12 months, laterality parameters were reduced but no other parameters changed significantly. This data suggests that digitomotography provides quantitative and objective measures capable to differentiate PD from non-PD in a small cohort, however, the value of the assessment to track PD progression has to be further evaluated in larger cohorts of patients.

## Introduction

Parkinson’s disease (PD) is a chronic and progressive neurodegenerative condition with motor and non-motor deficits. The Unified PD Rating Scale (UPDRS) is the primary outcome measure currently used in most clinical trials investigating PD therapeutics [1–3]. However the scale is semi-quantitative and subjective, and has low reliability in particular for the assessment of the index finger tapping task [4,5] which reflects bradykinesia. Bradykinesia encompasses slowness and decrease of movement amplitude and is often accompanied by arrhythmicity. As both bradykinesia and arrhythmicity occur early on in the disease and progresses continuously, they may be promising targets for the development of quantitative outcome measures. Quantitative and objective outcome measures with high sensitivity to change are of great interest as they may facilitate early proof-of-concept studies of novel neuromodulatory treatment approaches, which may soon be translated from preclinical to human studies.

Index finger tapping with “as large an amplitude and as fast movements as possible”, which reflects the instructions for the UPDRS assessment, has already been investigated quantitatively. Application of a digitized switch board identified tapping frequency and variability as measures discriminating between 51 PD early to mid-stage patients and 36 controls [6]. Another cross-sectional study investigating 50 PD patients with a mean Hoehn & Yahr (HY) stage of 2.4 [7] compared finger tapping sub-items of the UPDRS with quantitative parameters of frequency, amplitude and rhythmicity obtained from inertial sensors worn at the index finger and concluded that the sensors “can objectively measure speed, amplitude, and rhythm without reliability concerns associated with clinical rating scales”. Another study investigating index finger tapping in 40 PD patients with a mean HY of 2.3 and in 14 controls by use of a gyro sensor found highly significant differences of frequency and amplitude between the cohorts, and high correlation of these values with the UDPRS finger tap scores obtained by a neurologist [8]. Moreover, one study [9] investigated index finger tapping in 33 mildly to moderately affected PD patients, 21 controls and 18 patients with essential tremor by use of a small sensor placed over the distal interphalangeal joint of the index finger; interestingly the variability but not the frequency measure differed between PD patients and controls [9]. Indeed, the UPDRS seems to capture more the amplitude than the speed category: Amplitude impairment during index finger tapping was mirrored by the UPDRS total and UPDRS motor scores in 23 PD patients during Off state, but not the speed impairment [10].

These results indicate that quantitative and objective assessment of index finger tapping has indeed a potential to represent bradykinesia and may supplement the clinical assessment. However it has, to the best of our knowledge, not been investigated whether quantitative tapping measures differentiate among distinct stages of PD, and whether these parameters show relevant longitudinal changes over follow-up periods usually applied in clinical trials. By using a device and protocol that has been shown to be highly sensitive to change in preclinical and clinical phases of Huntington’s disease [11–13], this study thus aimed at comparing quantitative index finger tapping measures between three cohorts, i.e. early PD, mid-stage PD and controls, and to determine changes of these measures over a 12 months period.

## Methods

### Participants and clinical assessment

Sixteen PD patients with a disease duration between 0 and 3 years (defined here as “early PD”), 17 PD patients with a disease duration between 5 and 10 years (“mid-stage PD”) according to the UKPDS Brain Bank criteria [14] and 18 healthy control individuals were recruited from the outpatient clinic at the Neurodegenerative Department of the University of Tuebingen, Tuebingen, Germany. Participants had to achieve a total score of at least 25 points in the Mini Mental State Examination (MMSE [15]). Initially we tried to measure all patients during Off medication which was not possible due to logistic reasons (see also Table 1). Spouses of the included PD patients who did not have a history of neurological diseases were asked to serve as controls. All individuals underwent clinical testing including the motor part of the revised version of the UPDRS [16]), the HY Scaling [17], the Trail Making Test (TMT, where we then calculated Delta TMT = part B minus part A. Delta TMT is considered a measure of cognitive flexibility and working memory [18]) and the Becks Depression Inventory (BDI [19]). All participants were re-assessed after 12 months with the identical assessment protocol.

**Table 1:** Demographics and clinical characteristics.

	Controls	Early PD	Mid-stage PD	*P*-value
Participants (females)	18 (8)	16 (8)	17 (7)	0.88
Age [ys]	67 (50–75)	65 (50–70)	67 (56–76)	0.11
Age at disease onset [ys]		63 (48–69)	60 (51–71)	0.99
Disease duration [ys]		1.6 (0–3)	6.7 (5–9)	<0.0001
Subtypes (tremor-dominant/indeterminate/akinetic-rigid)		1/9/5	0/13/4	0.35
Hoehn & Yahr stage (1–5)		2 (1–2)	3 (1–4)	<0.0001
Levodopa dose equivalency		213 (0–540)	764 (310–1440)	<0.0001
On / Off state		6/10	3/14	0.20
UPDRS III (0–132)	2 (0–4)	20 (4–32) °	32 (8–68) ° *	<0.0001
UPDRS finger tapping (0–8)	0 (0–1)	3 (0–6) °	3 (0–7) °	<0.0001
MMSE (0–30)	29 (27–30)	29 (27–30)	29 (25–30)	0.52
BDI (0–63)	3 (0–10)	6 (0–15)	9 (2–31) °	0.001
Education [ys]	10 (9–13)	10 (9–13)	10 (9–13)	0.71

Data are presented with median (range) and frequency. Statistical comparisons were performed with the Kruskal-Wallis / Wilcoxon rank sum test, and the Pearson / Fisher’s Exact test, with analyses between single cohorts using Bonferroni correction (controls versus early PD, controls versus mid-stage PD, early PD versus mid-stage PD, *p* < 0.05/3 = 0.017).

° Compared to controls

* Compared to early Parkinson’s disease (PD).

BDI, Becks Depression Inventory; MMSE, Mini-Mental State Examination; UPDRS III, Motor part of the Unified Parkinson’s Disease Rating Scale.

### Ethics statement

The study was approved by the local ethical committee of the Medical Faculty of the University of Tuebingen, Germany, and all participants gave their written informed consent. Performance of the study was according to the principles of the Declaration of Helsinki.

### Digitomotography-index finger tapping

**Fig 1:**
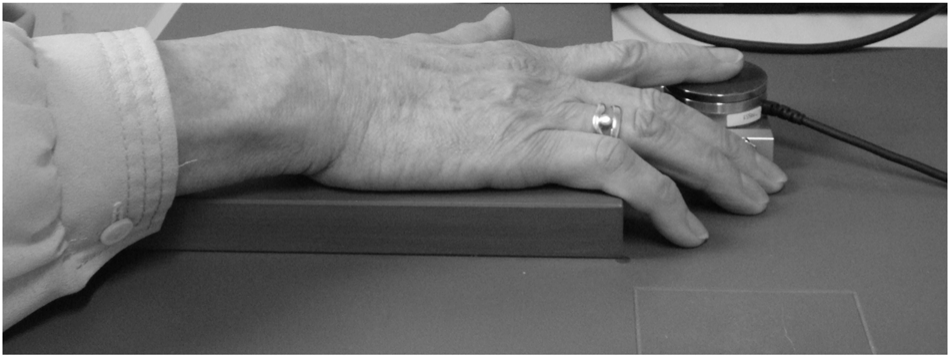
Q-Motor digitomotography device and position of the hand for the index finger tapping assessment. The hand with palm down is placed on a fixed support surface on a table, with the index finger located above the force transducer surface before the tapping experiments are started.

Index finger tapping was assessed using the “digitomotography” setup of the quantitative motor (Q-Motor) battery, using a pre‐calibrated and temperature controlled force transducer with a circular plane contact surface measuring 40 mm in diameter (Mini‐40, ATI Industrial Automation, Apex, NC, USA) [11]. Study participants were asked to place the respective hand palm down on a support surface in front of them on a table, with the index finger located above the force transducer surface such that they could comfortably tap on the sensor (Fig 1). They were then asked to perform 3 trails of tapping “as fast and regular as possible” for 10 seconds, as well as 3 trials of metronome paced tapping. Beginning and cessation of a trial was indicated by a beep. At the beginning of the metronome paced tapping, 10 consecutive rhythmic cueing tones with a frequency of 1.55 Hertz were presented and the participants were asked to match this rhythm, and then to continue with this frequency for another 10 seconds without cueing. These latter ten seconds were recorded and analyzed. Sampling frequency was 400 Hertz. Quantitative data were stored on a laboratory computer system (WinSCP/WinZoom, University of Umeå, Umeå, Sweden).

### Data processing and extraction of tapping parameters

Processing and analysis of tapping data was performed blinded at the GHI in Muenster using semi-automated software after quality control. The following parameters were extracted, and provided for the left and right side separately: mean and standard deviation (SD) of the inter-peak-interval-IPI (IPI mean, IPI SD), mean and coefficient of variation of tap force-TF (TF mean, TF CoV), and mean and variability of tap deviation-DEV (DEV mean, DEV SD) from the predefined 1.55 Hertz cueing tone. These six parameters were chosen as they most probably reflect – at least partly-different aspects of motor deficits (in particular of bradykinesia and rigidity) associated with PD: IPI mean as a measure of frequency, TF mean as an indirect measure of amplitude, DEV mean as a measure of tap deviation, IPI SD as a measure of arrhythmicity of speed, TF CoV as an indirect measure of variation of amplitude, and DEV SD as a measure of variation of tap deviation.

### Statistical analysis

Extracted data were analyzed with JMP statistical software (Version 9.0.2, SAS Institute Inc., Cary, NC, USA). Demographic and clinical data are presented with either median (range) or frequency (percent of total), and were calculated using non-parametric tests (Wilcoxon rank sum, Kruskal-Wallis tests) or Pearson / Fisher’s Exact test. Quantitative tapping data was normally distributed and therefore calculated using parametric test procedures (ANOVA, Student’s t test). Except for laterality, averaged values derived from the left and right index finger were used for comparisons between cohorts. Laterality was defined by using the formula |right – left|. Simple regression analysis and the determination coefficient (r²) were used to determine the strength of association between quantitative tapping parameters and clinical variables. For the calculation of longitudinal data, the early PD and the mid-stage PD cohort were merged because we felt that clinically meaningful changes should be detectable independent of disease stage.

A two-tailed approach was used. *P*-values below 0.05 were considered statistically significant. Bonferroni correction (*p* < 0.05/3 = 0.017) was applied where appropriate (Tables 1 and 2; significant difference between early PD and controls, mid-stage PD and controls, as well as early PD and mid-stage PD in case of significant p-value in ANOVA). We did not correct for the six quantitative parameters included, as this part of the study can be defined as hypothesis-generating.

**Table 2:** Cross-sectional comparison of digitomotography measures between early patients with PD, mid-stage PD and controls.

	Controls	Early PD	Mid-stage PD	*P*-value
Averaged values				
IPI mean [s]	0.28 (0.05)	0.32 (0.12)	0.29 (0.07)	0.15
IPI SD [s]	0.024 (0.01)	0.044°° (0.03)	0.041° (0.03)	0.03
TF mean [N]	2.68 (1.95)	1.30 (0.62) °°	2.00 (1.12)	0.009
TF CoV	0.23 (0.06)	0.30 (0.13)	0.27 (0.09)	0.06
DEV mean [s]	0.027 (0.02)	0.049 (0.05)	0.064 (0.07) °°	0.046
DEV SD [s]	0.048 (0.01)	0.061 (0.02) °°	0.058 (0.02) °	0.03
Lateralization				
IPI mean [s]	0.030 (0.037)	0.078 (0.113)	0.059 (0.062)	0.095
IPI SD [s]	0.014 (0.018)	0.018 (0.025)	0.035 (0.059)	0.12
TF mean [N]	0.65 (0.46)	0.53 (0.46)	0.53 (0.54)	0.35
TF CoV	0.036 (0.026)	0.088 (0.075) °	0.104 (0.094) °°	0.008
DEV mean [s]	0.016 (0.017)	0.035 (0.050)	0.021 (0.017)	0.10
DEV SD [s]	0.012 (0.011)	0.025 (0.024) °°	0.010 (0.009) **	0.008

Data are presented with mean (standard deviation). Statistical comparisons were performed with ANOVA (right column). Results of analyses between single cohorts (controls versus early Parkinson’s disease (PD), controls versus mid-stage PD, early PD versus mid-stage PD) are displayed as follows: Compared to controls without ° and with °° Bonferroni correction (*p* < 0.05/3 = 0.017).

** Compared to early PD with Bonferroni correction.

CoV, coefficient of variation; DEV mean, mean tap deviation from the predefined 1.55 Hertz cueing tone; DEV SD, variability of tap deviation from the predefined 1.55 Hertz cueing tone; IPI, interpeak interval; SD, standard deviation; TF, tap force.

## Results

Table 1 gives an overview of demographic and clinical data. Early and mid-stage PD patients as well as control subjects were comparable with respect to age, gender and MMSE scores. As expected, PD patients differed from controls in H&Y and UPDRS motor scores, and mid-stage PD patients took higher dopaminergic doses than the early PD patients. Depressive symptoms were more often reported by mid-stage PD patients than by controls. Frequency of subtypes, and frequency of On / Off state did not significantly differ among the PD cohorts. As expected, UPDRS motor scores increased significantly from baseline to follow up in PD patients, compared to controls (p = 0.006), however MMSE, Delta TMT and BDI values remained stable during the observation period (p≥0.28).

### Cross-sectional comparisons of index finger tapping parameters between PD patients and controls

Table 2 provides details about the cross-sectional results. The following averaged index finger tapping parameters were significantly different between cohorts: IPI SD, TF mean, DEV mean and DEV SD. Analyses between single cohorts showed significant differences of IPI SD, TC mean and DEV SD between early PD patients and controls. Mid-stage PD patients differed from controls in DEV mean. Mid-stage PD patients also had higher DEV SD than controls, however this difference did not survive Bonferroni correction. None of the investigated parameters differed significantly between early and mid-stage PD.

Lateralization of TF CoV and DEV SD was significant after ANOVA. Analyses between single cohorts revealed that early PD patients had higher TF CoV than controls which approached significance after Bonferroni correction, and mid-stage PD had even higher TF CoV which was significant compared to controls. DEV SD was significantly higher in early PD compared to both, controls and mid-stage PD. This latter parameter was the only one which differed significantly between early and mid-stage PD, but was comparable between mid-stage PD and controls (Table 2).

Table 3 gives an overview of correlations between Q-Motor finger tapping measures and clinical parameters. IPI SD and DEV mean correlated with both the UPDRS motor part and the UPDRS finger tapping score. Moreover, the TF CoV correlated with the UPDRS finger tapping score. Of note, the highest correlation was found between IPI SD and the Delta TMT. Relevant correlations of tapping parameters were neither observed with the MMSE nor with the BDI.

**Table 3:** Correlation of digitomotography measures with clinical data.

	UPDRS III	UPDRS Fingertapping	MMSE	Delta TMT	BDI
IPI mean	0.02	0.01	0.02	0.07	0.03
IPI SD	0.15*	0.13*	0.06	0.25*	0.08*
TF mean	0.02	0.03	0.00	0.05	0.00
TF CoV	0.05	0.12*	0.00	0.00	0.02
DEV mean	0.16*	0.16*	0.05	0.05	0.06
DEV SD	0.07	0.06	0.00	0.03	0.01

Data are calculated with simple regression, and presented with the coefficient of determination (r^2^).

* *p* < 0.05.

BDI, Becks Depression Inventory; CoV, coefficient of variation; DEV mean, mean tap deviation from the predefined 1.55 Hertz cueing tone; Delta TMT, Trail Making Test part B minus part A, a measure of cognitive flexibility and working memory [18]; DEV SD, variability of tap deviation from the predefined 1.55 Hertz cueing tone; IPI, interpeak interval; MMSE, Mini-Mental State Examination; SD, standard deviation; TF, tap force; UPDRS III, Motor part of the Unified Parkinson’s Disease Rating Scale.

### Longitudinal analyses of index finger tapping parameters

Table 4 gives an overview of the longitudinal changes of the Q-Motor tapping measures. When corrected for control values, none of the averaged parameters of the PD patients showed significant changes to baseline. The following control-corrected lateralization parameters of PD patients decreased significantly from baseline to follow-up: IPI mean (i.e. right/left differences in frequency decelerated), TF mean (right/left differences in tap force decelerated), and TF CoV (right/left differences in variability of tap force diminished).

**Table 4:** Comparison of digitomotography parameters between baseline and 12 months follow-up assessments.

	Controls	PD	*P*-value
Averaged values			
IPI mean [s]	-0.017 (0.054)	-0.036 (0.114)	0.27
IPI SD [s]	0.005 (0.025)	-0.008 (0.030)	0.08
TF mean [N]	-0.45 (1.38)	0.08 (0.75)	0.05
TF CoV	0.023 (0.042)	-0.006 (0.072)	0.07
DEV mean [s]	0.066 (0.061)	0.101 (0.105)	0.12
DEV SD [s]	-0.001 (0.016)	-0.002 (0.015)	0.49
Lateralization			
IPI mean [s]	0.020 (0.081)	-0.032 (0.105)	0.048
IPI SD [s]	0.007 (0.050)	-0.007 (0.051)	0.19
TF mean [N]	0.52 (1.44)	-0.04 (0.63)	0.04
TF CoV	0.048 (0.043)	-0.015 (0.103)	0.01
DEV mean [s]	0.002 (0.025)	0.004 (0.055)	0.45
DEV SD [s]	0.001 (0.018)	-0.003 (0.024)	0.30

Data are presented with mean (standard deviation). Early and mid-stage PD cohorts were merged to increase statistical power. Statistical comparisons were performed with Student’s t test. CoV, coefficient of variation; DEV mean, mean tap deviation from the predefined 1.55 Hertz cueing tone; DEV SD, variability of tap deviation from the predefined 1.55 Hertz cueing tone; IPI, interpeak interval; SD, standard deviation; TF, tap force.

## Discussion

To the best of our knowledge, this is the first study investigating the potential of speeded and metronome paced index finger tapping – a classical test associated with bradykinesia in PD patients and included in the UPDRS – to detect cross-sectional differences between different stages of PD and assess longitudinal change of these measures. The study revealed that (i) PD patients can indeed be differentiated from controls when assessing fine motor function with a quantitative method such as the Q-Motor digitomotography test, (ii) some parameters may show U-shaped changes during the course of the disease, (iii) tapping parameters of variability (IPI SD and DEV SE, i.e. the tap deviation from a predefined rhythmic cueing tone) were associated with the UPDRS motor part, and (iv) the most promising symptom (or sign) for the assessment of longitudinal changes of fine motor function in PD may be lateralization.

This study basically confirms findings from previous cross-sectional studies [7–9] applying comparable assessments and also including early to mid-stage PD patients. Our results are particularly in agreement with [9] that described a significantly different IPI variability in their PD cohort compared to controls. Our study extends the findings of an increased variability of finger tapping in PD patients compared to controls insofar as the two parameters that assess regularity and variability of tapping after a metronome paced cue (DEV mean and DEV SD) were higher in both early and mid-stage PD, than in controls. Loss of rhythmicity and increase of variability during repetitive movements is a well-known characteristic of PD, and includes also axial movements such as gait [20]. We could not confirm that early to mid-stage PD patients have significantly lower tapping frequencies compared to controls as described in other studies investigating index finger tapping quantitatively [6,8,10]. As the above study [9] also did not find relevant frequency differences between PD and controls, we conclude that frequency may not be a promising marker for differentiating (early) PD patients from controls.

Some studies found lowered tapping amplitudes in early to mid-stage PD patients which has been associated with the degree of bradykinesia [8,9]. Although not directly assessed in this study, amplitude may at least partly be reflected by tap force. Average tap force was clearly reduced in our early PD cohort; interestingly, it was not significantly different between mid-stage PD and controls. This supports the hypothesis that parameters defining motor (dys)function during the course of a neurodegenerative disease may not always follow a linear curve but may “deviate” from a linear deterioration due to e.g. compensation mechanisms which indeed strongly interfere with continuously progressing deficits [21,22].

Lateralization of variability was more prominent in early PD than in mid-stage PD in the cross-sectional part of this study. Comparably, lateralization of the TF CoV showed a relevant change (reduction) in the longitudinal assessment. This was accompanied by a significant decrease of lateralization of TF mean. Laterality in PD using quantitative assessment tools has not been investigated extensively. One study assessed the mean velocity of repetitive alternating index and middle finger tapping in 85 PD patients with a musical keyboard, and found that the difference of tapping velocity between the more and less affected side decreased with increasing disease severity [23]. A clinical study investigating more than 1.000 PD patients found a significant negative association between lateralization of symptoms and disease duration [24]. Together with these previous results, the outcome of the longitudinal finger tapping assessments performed here suggests that (loss of) lateralization is the most promising target for the detection of progression of fine motor dysfunction in PD.

Comparable to previous studies [7–9] quantitative index tapping parameters correlated significantly with the UPDRS motor part and the finger tapping items of the UPDRS, arguing for the validity of this approach. We also confirmed the low correlation of frequency (i.e. IPI mean) with UPDRS ratings as previously demonstrated [10]. Interestingly, the highest correlation between Q-Motor tapping measures and clinical measures was found between the averaged IPI SD – a measure of tapping variability – and the Delta TMT which measures cognitive flexibility and working memory [18]. This result argues for an influence of executive function on rhythmicity even in very simple motor tasks; this aspect needs further exploration.

We acknowledge that our study has several limitations. Due to logistic difficulties, we have not assessed all patients in the Off state as stated above. Testing was not randomized and we did not assess fatigue, an important non-motor PD symptom. These two factors and the combination of these two factors could have a relevant influence on our results. Moreover, cohort sizes were rather small not permitting a meaningful analysis of the possible impact of medication on the effects observed. However, exclusion of patients in On state from the analyses did not relevantly influence the results (not shown). In spite of these limitations, cross-sectional and longitudinal effects could be observed.

In conclusion, results of this study confirm previous findings that digitomotography can indeed differentiate PD patients from controls. However, tracking PD progression using simple measures such as frequency or tap interval variability seems to be difficult. Results from this study suggest that the determination of (changes of) lateralization may be a promising approach to assess changes of motor symptoms during disease course. Importantly, the study demonstrates the well-established paradigm that cross sectional findings do not predict the longitudinal behavior of any measure and calls for the conduct of well-controlled prospective, multi-center biomarker studies in PD assessing quantitative motor and other endpoints in larger cohorts of patients.
